# Gene and Blood Analysis Reveal That Transfer from Brackish Water to Freshwater Is More Stressful to the Silverside *Odontesthes humensis*

**DOI:** 10.3389/fgene.2018.00028

**Published:** 2018-02-06

**Authors:** Tony L. R. Silveira, Gabriel B. Martins, William B. Domingues, Mariana H. Remião, Bruna F. Barreto, Ingrid M. Lessa, Lucas Santos, Danillo Pinhal, Odir A. Dellagostin, Fabiana K. Seixas, Tiago Collares, Ricardo B. Robaldo, Vinicius F. Campos

**Affiliations:** ^1^Laboratory of Structural Genomics, Technological Development Center, Federal University of Pelotas, Pelotas, Brazil; ^2^Laboratory of Physiology, Institute of Biology, Federal University of Pelotas, Pelotas, Brazil; ^3^Laboratory of Cancer Biotechnology, Technological Development Center, Federal University of Pelotas, Pelotas, Brazil; ^4^Genomics and Molecular Evolution Laboratory, Department of Genetics, Institute of Biosciences of Botucatu, São Paulo State University, Botucatu, Brazil; ^5^Laboratory of Vaccinology, Technological Development Center, Federal University of Pelotas, Pelotas, Brazil

**Keywords:** acclimation, blood, brackish water, fish, freshwater, genes, salt, transfer

## Abstract

Silversides are fish that inhabit marine coastal waters, coastal lagoons, and estuarine regions in southern South America. The freshwater (FW) silversides have the ability to tolerate salinity variations. *Odontesthes humensis* have similar habitats and biological characteristics of congeneric *O. bonariensis*, the most studied silverside species and with great economic importance. Studies revealed that *O. bonariensis* is not fully adapted to FW, despite inhabiting hyposmotic environments in nature. However, there is little information about stressful environments for cultivation of silverside *O. humensis*. Thus, the aim of this study was to evaluate the stress and osmoregulation responses triggered by the osmotic transfers on silverside *O. humensis*. Silversides were acclimated to FW (0 ppt) and to brackish water (BW, 10 ppt) and then they were exposed to opposite salinity treatment. Silverside gills and blood were sampled on pre-transfer (D0) and 1, 7, and 15 days (D1, D7, and D15) after changes in environmental salinity, the expression levels of genes *atp1a3a*, *slc12a2b*, *kcnh1*, and *hspa1a* were determined by quantitative reverse transcription-PCR for evaluation of osmoregulatory and stress responses. Furthermore, glycemia, hematocrit, and osmolality were also evaluated. The expression of *atp1a3a* was up- and down-regulated at D1 after the FW–BW and BW–FW transfers, respectively. *Slc12a2b* was up-regulated after FW–BW transfer. Similarly, *kcnh1* and *hspa1a* were up-regulated at D1 after the BW–FW transfer. *O. humensis* blood osmolality decreased after the exposure to FW. It remained stable after exposure to BW, indicating an efficient hyposmoregulation. The glycemia had a peak at D1 after BW–FW transfer. No changes were observed in hematocrit. The return to the pre-transfer levels at D7 after the significant increases in responses of almost all evaluated molecular and blood parameters indicated that this period is enough for acclimation to the experimental conditions. In conclusion, our results suggest that BW–FW transfer is more stressful to *O. humensis* than FW–BW transfer and the physiology of *O. humensis* is only partially adapted to FW.

## Introduction

*Odontesthes* spp., popularly known as pejerreyes or silversides, is a fish genus naturally endemic from South America waters (Bemvenuti, 2006). This genus comprises the biggest number of species of Atherinopsidae and the broader area of distribution, with species inhabiting marine coastal waters, coastal lagoons, and estuarine regions in southern South America (Dyer, 2006). Currently, some fishes from these species occupy FW environments, despite all *Odontesthes* spp. have a recent common marine origin (Heras and Roldán, 2011; Campanella et al., 2015). Thus, the FW silversides have an interesting ability to tolerate salinity variations. This euryhaline characteristic has drawn attention of researchers to its application in aquaculture in estuarine regions, where there are continuous alterations in water salinity (Piedras et al., 2009).

The most studied species of silverside, with economic importance and favorable projections for production, is *Odontesthes bonariensis*. In Argentina, it has an importance for flesh commercialization and sport fishing (Menone et al., 2000; Somoza et al., 2008). In Japan, *O. bonariensis* cultivation has been deployed, due to the efforts to farming conditions improvements in the last few years (Tsuzuki et al., 2000b, 2001, 2007). This species has also been introduced in Chile, Bolivia, and Italy (Dyer, 2000, 2006). The species is also exotic and has economic importance in Peru, where *O. bonariensis* was introduced in the Titicaca Lake in 1946 and until nowadays continues to be caught (Loubens and Osorio, 1991).

On the other hand, the congeneric *Odontesthes humensis* receives little attention from researchers and fish farmers. Only studies involving fry growth (Pouey et al., 2012), embryos salinity tolerance (Piedras et al., 2009), growth in intensive system (Tavares et al., 2014), and toxicology (Zebral et al., 2017) are developed in captivity specimens. Both *O. bonariensis* and *O. humensis* species have similar habitats, biological characteristics (including morphology), and are capable of interbreeding. Due to this, both species have similar potential for captivity cultivation (Dyer, 2006; Piedras et al., 2009). Main differences between both species are: *O. bonariensis* is the largest species in Atheriniforms and it has 32–38 gill rakers on lower branch, vomerine teeth present, and the prognathism can be present. Furthermore, this species is native to Brazil and Argentina, but not to Uruguay. On the other hand, *O. humensis* is smaller, has 13–19 gill rakers on lower branch, vomerine teeth is absent, prognathism is never present, and the species is native to Brazil, Uruguay, and Argentina (Dyer, 2006). Although the commercial production of *O. humensis* has not increased in recent years, this species has potential to be a bioindicator in toxicological studies (Zebral et al., 2017) due to its demands for water quality, surviving only in a narrow range of water parameters.

Thus, information about basic biology of *O. humensis* is necessary to the adequate maintenance of this silverside species in farm and laboratory conditions. Some studies revealed that the silverside *O. bonariensis* is not fully adapted to FW yet, despite inhabiting hyposmotic environments in nature (Tsuzuki et al., 2000b, 2001). However, there is no information about the less stressful environment, if with or without salt, to the cultivation of silverside *O. humensis*. We hypothesized that *O. humensis* shared, besides the habitats and reproductive physiology, the same pattern of response of *O. bonariensis* to environmental salinity. The corroboration of this hypothesis will help in maintenance of laboratorial or commercial cultivation, in addition to reinforcing the theory of invasion of FW habitats by silversides marine ancestors. Thus, the aim of this study was to evaluate, using cellular, biochemical, and molecular analysis, the stress and osmoregulation responses triggered by the osmotic transfers in silverside *O. humensis*.

## Materials and Methods

### Animals and Conditions

The silversides *O. humensis* used in this study came from eggs collected in nature (Arroio Grande, Brazil – 32°14′15^′′^S/53°05′13^′′^W) and hatched in tanks. The eggs were collected from wild breeders and incubated at laboratory conditions in FW (0) and pH values near 7.5, the same conditions applied to larviculture. The fish were 1.5 years old and have mean weight of 27.3 ± 9.5 g at experimental time. The silversides were maintained in 1,000 L cylindrical plastic tanks within an experimental room, fed three times a day with commercial feed (Supra, 38% crude protein) until satiety, under natural photoperiod of autumn (11 h light/13 h dark), with water pH 7.5 ± 0.3, and 19 ± 0.3°C for acclimation. The tank sides were opaque to reduce visual stress and incidence of luminosity through the top according to the daily variance. The acclimation period was 4 weeks for silversides in FW (salinity of 0 ppt) and in BW (salinity of 10 ppt), under the same previous conditions. The salinity levels were achieved through the dissolution of non-iodized sea salt in the water.

The fish continued to be fed three times a day during the experimental period. Once a week the water of the tanks was totally renewed with dissolved oxygen maintained in 7.8 ± 0.3 mg L^-1^; the total ammonia levels lower than 0.6 ± 0.2 mg L^-1^; temperature 20.01 ± 0.34°C; and pH 7.78 ± 0.03.

### Experimental Design and Protocol

The experimental setup consisted in two treatments performed in quadruplicate, totaling eight tanks with 15 fish each. Four tanks contained silversides acclimated to FW and other four tanks contained silversides acclimated to BW. Both FW and BW groups were shortly transferred to opposite salt treatment. Fish were sampled at four different time points getting three fish per tank: a control before the osmotic transfer (D0) and on day 1 (D1), on day 7 (D7), and on day 15 (D15) after the transfer. Hence, 96 silverside fish were analyzed in different times of salt exposure.

### Sampling

When captured, at all time points, the silversides were anesthetized with benzocaine (50 mg L^-1^). Blood samples were collected from caudal puncture using a heparinized syringe for hematocrit, glycemia, and osmolality evaluation. While anesthetized, the fish were euthanized by medullary section and brain excision to perform the post-mortem gills collection. Gill samples from three fish of each tank, at the four time points, were collected using sterilized material. Left second branchial arches were collected and immediately stored in liquid nitrogen (N_2_) until posterior RNA preparation. All experimental procedures were previously approved by the Ethics Committee on Animal Experimentation of Federal University of Pelotas (Process no. 23110.007018/2015-85).

### Analyzed Genes

The Na^+^/K^+^-ATPase (NKA) enzyme is responsible to active transports three Na^+^ out of and two K^+^ into animal cells. In fishes, NKA is directly involved in the osmoregulation process (Hiroi and McCormick, 2012). Among NKAα1∼α4, we selected NKAα3 (encoded by *atp1a3*) which has similar responses (Stefansson et al., 2007; Whitehead et al., 2012; Armesto et al., 2014; Li et al., 2014; Taugbøl et al., 2014) and is expressed in similar tissues (Guynn et al., 2002) as NKAα1. NKAα1 is the most studied isoform of NKA (Havird et al., 2013), however, its mRNA expression can be influenced by the stress (Kiilerich et al., 2011) and this could disrupt the interpretation of results.

The Na^+^/K^+^/2Cl^-^ cotransporter 1 (NKCC1) mediates the intake of one Na^+^, one K^+^, and two Cl^-^ ions into the ionocytes down the electrochemical gradient generated by NKA (Hwang et al., 2011). In teleosts there are two isoforms of NKCC1 and NKCC2: NKCC1a/b and NKCC2a/b (Kakumura et al., 2015). NKCC1 (encoded by *slc12a2*) is more associated with ion secretion in teleost gills (Tipsmark et al., 2002; Nilsen et al., 2007), whereas NKCC2 mRNA is not present in large amount in fish gills (Hiroi and McCormick, 2012).

The voltage-gated potassium channels (KCNH) are gated by changes in membrane potential and play an important role in the repolarization of cells (Morais-Cabral and Robertson, 2015). KCNH1 has already been correlated to myoblast fusion (Occhiodoro et al., 1998), to embryonic development (Stengel et al., 2012), and has been used as target to cancer researches (Pardo and Stühmer, 2013) but the precise role of KCNH1 in normal tissues is poorly known. The *kcnh1* gene has two copies in fishes, *kcnh1a*/*b* that are expressed differently in the organism (Stengel et al., 2012). KCNH4 has already been proposed as an osmoregulation marker (Taugbøl et al., 2014).

The heat shock 70kDa proteins (HSP70) are mainly responsible to protein folding, degradation of misfolded proteins, membrane translocation, and activation of immune system. HSP70 are expressed under normal conditions but also in response to stress conditions (Morimoto, 1998; Yamashita et al., 2010; McCallister et al., 2016). The genes encoding HSP70 in fishes are *hspa1a* and *hspa1b* (He et al., 2013). Thus, both *hspa1* genes and the HSP70 protein are considered stress indicators in fish (Basu et al., 2001; Larsen et al., 2012; Taugbøl et al., 2014).

### Molecular Cloning and Sequencing

Total RNA was isolated from gill samples of each fish separately, using RNeasy^®^ Mini Kit (Qiagen, United States) according to the manufacturer’s protocol. DNase treatment of RNA samples was conducted with a DNA-Free^®^ Kit (Ambion, United States) following the manufacturer’s protocol. The RNA concentration and quality were verified by spectrophotometry using NanoVue^TM^ Plus (GE Healthcare Life Sciences, United states), and only samples with absorption *A*_260_/*A*_280_ ratio ≥2.0 were used in reverse transcription (RT) reactions.

First-strand cDNA was performed with 2 μg of RNA using random primers and SuperScript^®^ III Reverse Transcriptase (Invitrogen, United States) according to the manufacturer’s protocol. Primer sets (**Table 1**) were designed to clone partial cDNA sequences of *atp1a3*, *slc12a2*, *kcnh1*, and *hspa1* genes from gills of silverside fish based on the alignment of sequences of other fish species. The PCR parameters were: an initial denaturation for 1 min at 94°C, followed by 35 cycles of 94°C for 30 s; 57°C (for *atp1a3*); 62°C (for *slc12a2*); 64.6°C (for *kcnh1*); and 61°C (for *hspa1*) for 30 s; and 72°C for 1 min, with a final extension of 5 min at 72°C. PCR products were inserted into pCR^TM^4-TOPO^®^ TA cloning vector and transformed in the electrocompetent *Escherichia coli* strain DH5α. These procedures were performed according to the manufacturer specifications (Invitrogen, United States). The positive clones were sequenced using a Big Dye^®^ V3.1 Terminator Kit and M13 Primers (Applied Biosystems, United States). The cloned fragments were sequenced using an Applied Biosystems 3500 Genetic Analyzer^®^ automatic sequencer (Life Technologies, United States).

**Table 1:** Sequences of primers for *Odontesthes humensis* used in this study.

Gene symbol	Sequence 5′→3′	Use
*atp1a3a*		
	F: AACCCCAGAGATGCCAA	Cloning
	R: AAGGCACAGAACCACCA	
	F: ATACCGGGGCAGTAGGAGAG	qPCR
	R: CAGTATCGTGGTGGTGCAGT	
*slc12a2b*		
	F: GGRATGGARTTGGARGCHAARGC	Cloning
	R: CCDGABACCAGGCTCATBACCTG	
	F: GCCATCCTCATCACAGGATT	qPCR
	R: ACTGTGTCGCTCATCGTGTC	
*kcnh1*		
	F: CAGCACCTGCAGCTTCATG	Cloning
	R: CCACRATGCTGTCYACCACCAGCC	
	F: TTGTCGGGGTACCATAGAGC	qPCR
	R: AGTCGCACTTTTTCCGTTGT	
*hspa1a*		
	F: TCTGCAGCTAAAGGTGTAGC	Cloning
	R: TTGAAGGGCCAGTGCTTCATG	
	F: TCCAGCACGGAAAAGTAGAGA	qPCR
	R: ACAGTGTTGCTGGGGTTCAT	
*actb*		
	F: CTCTGGTCGTACCACTGGTATCG	qPCR reference gene
	R: GCAGAGCGTAGCCTTCATAGATG	
*h3a*		
	F: CGTGGCTCTGAGAGAGATCC	qPCR reference gene
	R: TCGGTCTTGAAGTCCTGAGC	

### *In Silico* and Phylogenetic Analysis

The identity of all sequenced cDNA was confirmed by Blast tool of the NCBI database^1^. The translation of sequenced nucleotides to amino acid sequences as well as the open-reading frame (ORF) identification was made using ExPASy bioinformatics resource portal^2^. Conserved domains and sites were mapped from UniProt database^3^. Amino acid sequences of NKA, NKCC, KCNH, and HSP70 from various vertebrate species were obtained from GenBank and aligned to the new deduced amino acid sequences of *O. humensis* using ClustalW. Phylogenetic analyses were carried out using MEGA version 6 (Tamura et al., 2013). Phylogenetic trees were constructed based on alignments of amino acid sequences using Neighbor-Joining method. Bootstrap analyses were done with 10,000 replications to test the tree reliability. The outgroups were *Caenorhabditis elegans* (for NKA and HSP70 analysis), *Strongylocentrotus purpuratus* (for NKCC analysis), and *Toxocara canis* (for KCNH analysis).

### Evaluation of Gene Expression after Salinity Changes

Quantitative RT-PCR (qRT-PCR) was run on CFX96^TM^ Real-Time PCR Detection System (Bio-Rad Laboratories, United States) using SYBR^®^ Green PCR Master Mix (Applied Biosystems, United States). Primers (**Table 1**) for the identified genes *atp1a3*, *slc12a2*, *kcnh1*, and *hspa1* and for the used reference genes β-actin (*actb*, GenBank Accession No. EF044319) and histone h3a (*h3a*, GenBank Accession no. KX060037) were designed using Primer3 online software^4^. Initial validation experiments were conducted to ensure that all primer pairs had equivalent PCR efficiencies calculated by standard dilution curve. Amplification was carried out at the standard cycling conditions of 95°C for 10 min, followed by 40 cycles at 95°C for 15 s, 60°C for 60 s followed by conditions to calculate the melting curve. All PCR runs for each cDNA sample were performed in triplicate. The qRT-PCR data were analyzed using the 2^-ΔΔC_t_^ method considering primer amplification efficiencies (Livak and Schmittgen, 2001).

### Blood Analysis

After blood collection, with fasted animals, the glycemia was measured using Accu Chek Glucometer (Roche Diagnostics, United Kingdom). The samples from each fish were immediately transferred to microcapillaries and centrifuged at 12,000 ×*g* for 5 min to hematocrit analysis. The blood was transferred to sterilized microtubes and centrifuged at 1,500 ×*g* at 4°C for 10 min to plasma separation for osmolality measurements in Vapro 5520^®^ Vapor Pressure Osmometer (Wescor, United States).

### Statistical Analysis

Quantitative data were expressed as means ± standard error of mean. Significant differences among means were evaluated by two-way ANOVA followed by Tukey’s test, setting the significance level at 95% (*P* < 0.05). The exception was the expression of reference genes, which was expressed as means of cycle threshold (Ct) values ± standard deviation of mean and the differences were analyzed by one-way ANOVA followed by Tukey’s test.

## Results

### cDNA Cloning and Characterization

The *atp1a3*, *slc12a2*, *kcnh1*, and *hspa1* cloned fragments from *O. humensis* were, respectively, 975, 423, 560, and 286 bp (base pairs) in length. They were sequenced and deposited under GenBank accession numbers KR920364, KT001464, KX035016, and KU639716, respectively. The cloned fragments of *atp1a3* and *kcnh1* belong, respectively, to the middle of the ORF +2 and to the initial part of the ORF +3. The fragments of *slc12a2* and *hspa1* are part of the center of ORF +1. The *atp1a3* fragment codes to 324 amino acids belonging to the P-type ATPase family. The *slc12a2* codes to 141 amino acid residues belonging to the Na/K/Cl co-transporter 1 family. The cloned fragment of *kcnh1* codes to 186 amino acids belonging to the potassium channel, voltage-dependent family. The cloned fragment of *hspa1* codes to 95 amino acids belonging to the heat shock protein 70 family.

The percentage identity values between NKAα3, NKCC1, KCNH, and HSP70 putative sequences of silverside and of the other analyzed species were, respectively, 77–98, 55–92, 26–99, and 37–97% (**Supplementary Figures S1**–**S4**). The phylogenetic tree constructed based on alignment of NKAα-subunits amino acid sequences (**Supplementary Figure S1**) reveals that α1, α2, α3, and α4 form different clusters with mammal and fish sequences in each, except in α4 group, which was devoid of NKA sequences of fishes. Furthermore, the silverside NKAα3 was grouped in the monophyletic α3 cluster, showing that it was more closely related to NKAα3a than NKAα3b and NKAα2. Based on these evidences, the sequence found in the gills of *O. humensis* was identified as *atp1a3α*. NKCC1 and NKCC2 were grouped in different clusters (**Supplementary Figure S2**).

The paralogous NKCC1a and NKCC1b were grouped together in NKCC1 group. Furthermore, the tree has shown more evidence that silverside NKCC is closer to fish NKCC1b than to NKCC1a or NKCC2. Thus, the cloned and sequenced cDNA from silverside gills was identified as *slc12a2b*. The KCNH phylogenetic tree (**Supplementary Figure S3**) forms two monophyletic main groups: ELK family cluster, composed by vertebrates KCNH3, KCNH4, and KCNH8, and EAG family cluster, composed by vertebrates KCNH5 and KCNH1, in which the new sequence of silverside KCNH1 was grouped. Based on this analysis, the cloned KCNH sequence from silverside gills was confirmed as *kcnh1*.

The HSP70 phylogenetic analysis (**Supplementary Figure S4**) separated HSPA1 and HSPA2 sequences in different clusters. HSPA2 was composed only by sequences of mammals while HSPA1 was composed by mammals and teleosts. The new sequence of HSPA1 from silverside was clustered within fish HSPA1a. Based on these results, the HSPA sequence from silverside gills was identified as *hspa1a*.

### Gene Expression after Salinity Changes

**FIGURE 1:**
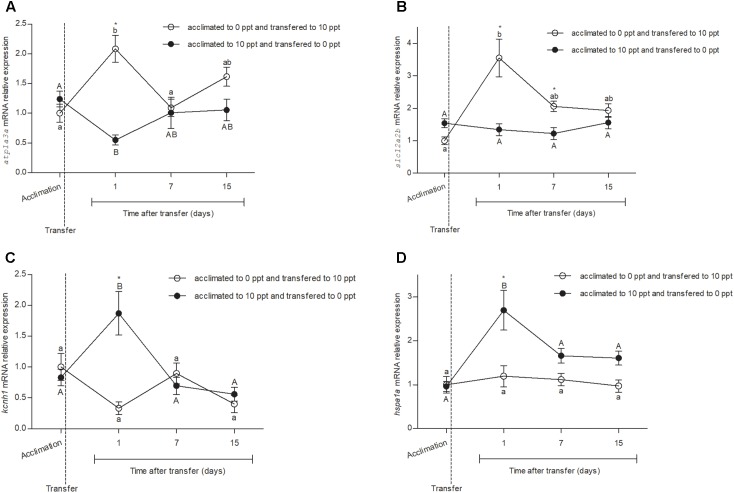
Relative expression of cloned genes *atp1a3a*, *slc12a2b*, *kcnh1*, and *hspa1a* from *Odontesthes humensis* submitted to salinity transfer. **(A)** *Atp1a3a* mRNA relative expression before and after the salinity change. **(B)** *Slc12a2b* mRNA relative expression before and after the salinity change. **(C)** *Kcnh1* mRNA relative expression before and after the salinity change. **(D)** *Hspa1a* mRNA relative expression before and after the salinity change. Mean ± SEM (*n* = 8–10). Different letters in each group (lowercase to FW–BW transferred group and uppercase to BW–FW transferred group) represent significant difference between the measurements in a same group. The asterisk represents the significant difference between groups in a same experimental time. The vertical broken line indicates the transfer from BW to FW and from FW to BW moments.

Primer sets for *atp1a3a*, *slc12a2b*, *kcnh1*, *hspa1a*, *actb*, and *h3a* had amplification efficiencies in qRT-PCR of 1.14, 1.07, 1.06, 1.02, 0.98, and 1.05, respectively. No difference was observed in the expression of reference genes between four different time points or between the treatments (**Supplementary Table S1**). The relative expression of *atp1a3a* (**Figure 1A**) was not different (*P* > 0.05) between FW- and BW-acclimated fishes in control D0. However, after the transfer of FW-acclimated fish to BW (FW–BW) and vice versa (BW–FW), a change in *atp1a3a* expression was observed. In FW–BW-transferred fish, there was an increase (*P* < 0.05) in *atp1a3a* expression, while in BW–FW there was a decrease at D1 (*P* < 0.05). The levels of *atp1a3a* expression returned close to the initial conditions in both FW–BW and BW–FW groups, without difference between groups (*P* > 0.05), at D7 and D15.

The relative expression of *slc12a2b* (**Figure 1B**) was not different between FW- and BW-acclimated fish at D0 (*P* > 0.05). However, after the FW–BW and BW–FW transfers, a change in *slc12a2b* mRNA expression was observed. In FW–BW-transferred fish, there was an increase (*P* < 0.05) in *slc12a2b* mRNA levels at D1. The gene expression decreased (*P* < 0.05) to intermediary levels at D7 and remained without difference until D15 (*P* > 0.05). The *slc12a2b* expression levels did not change (*P* > 0.05) after the transfer in the BW–FW group in any experimental time. Difference in *slc12a2b* relative expression between the BW–FW and FW–BW groups (*P* < 0.05) was observed at D1 and D7.

None significant difference was observed between the relative expression of *kcnh1* (**Figure 1C**) at D0 from FW- or BW-acclimated silversides (*P* > 0.05). After the transfers, at D1, it was observed a significant increase (*P* < 0.05) in the *kcnh1* expression of the BW–FW group. The expression levels decreased (*P* < 0.05), returning to the initial patterns at D7 and D15. The *kcnh1* expression levels did not change significantly (*P* > 0.05) after the transfer in the FW–BW group in any experimental time. Difference in *kcnh1* relative expression between the BW–FW and FW–BW groups (*P* < 0.05) was observed only at D1.

The relative expression of *hspa1a* (**Figure 1D**) was not different between FW- and BW-acclimated fish at D0 (*P* > 0.05). After the transfers, at D1, it was observed a significant increase (*P* < 0.05) in the *hspa1a* relative expression of the BW–FW-transferred fish. The expression levels decreased (*P* < 0.05) to initial pattern at D7 and remained unchanged until D15 (*P* > 0.05). The *hspa1a* expression levels did not change (*P* > 0.05) after the transfer in the FW–BW group in any experimental time. Difference in *hspa1a* relative expression between the BW–FW and FW–BW groups (*P* < 0.05) was observed only at D1.

### Glycemia

**FIGURE 2:**
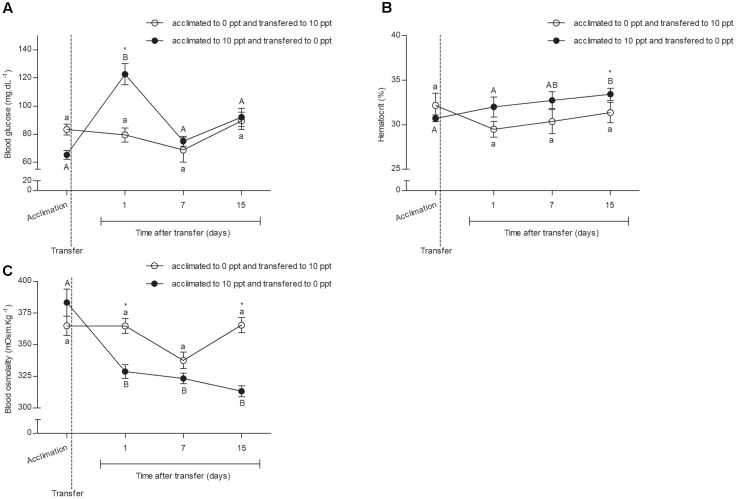
Blood responses from *O. humensis* submitted to salinity transfer. **(A)** Glycemia before and after the salinity change. **(B)** Hematocrit before and after the salinity change. **(C)** Blood osmolality before and after the salinity change. Mean ± SEM (*n* = 12). Different letters in each group (lowercase to FW–SW transferred group and uppercase to SW–FW transferred group) represent significant difference between the measurements in a same group. The asterisk represents the significant difference between groups in a same experimental time. The vertical broken line indicates the transfer from SW to FW and from FW to SW moments.

No difference in blood glucose concentration (*P* > 0.05) was observed between FW- or BW-acclimated fish at D0 (**Figure 2A**). The BW–FW-transferred fish presented an increase (*P* < 0.05) in glycemia values at D1, a decrease (*P* < 0.05) to pre-transfer levels at D7, and remained without difference (*P* > 0.05) until D15. None difference (*P* > 0.05) between time-points was observed in FW–BW transfer group. Significant difference between both groups was observed only at D1.

### Hematocrit

No difference (*P* > 0.05) between the hematocrit (**Figure 2B**) in none group of analyzed silverside of experimental time was observed. Only trends of increase in hematocrit soon after BW–FW transfer and decrease after FW–BW were verified.

### Osmolality

No difference (*P* > 0.05) between the blood osmotic concentration (**Figure 2C**) of FW- and BW-acclimated fishes was observed at D0. After transfer, there was a reduction (*P* < 0.05) in osmolality of BW–FW-transferred fish that was kept the same until D15. Differences were not observed in blood osmolality of FW–BW-transferred fish. Differences (*P* < 0.05) between groups were found right after the transfer (D1) and at the final measurements (D15).

## Discussion

Salinity is a frequent abiotic stressor that restrains fish growth and development by inducing osmotic stress responses. When fishes are subjected to salinity stress, related genes are activated to induce salinity stress tolerance. This study was successful in the identification and characterization of *atp1a3a*, *slc12a2b*, *kcnh1*, and *hspa1a* mRNA sequences in the silverside *O. humensis*. The molecular cloning made possible to adequately monitor changes in the expression of these genes after salinity challenges. In addition, it was demonstrated that alterations in blood glucose and osmolality after environmental salinity change.

In FW–BW-transferred fish, *atp1a3a* expression increased soon upon salinity stress, whereas in BW–FW group it was downregulated. Similar effects in *atp1a3a* expression were also observed in gills of Mozambique tilapia challenged by salinity stress, which presented higher expression levels in SW and lower in FW (Li et al., 2014). In threespine stickleback, the SW transfer also generated an increase in *atp1a3a* mRNA expression (Taugbøl et al., 2014). The increase of mRNA expression and protein activity of NKAα-subunit in gills, in response to the SW transfer, occurred in Atlantic salmon (D’Cotta et al., 2000), in brown trout (Tipsmark et al., 2002), and in killifish (Scott and Schulte, 2005; Scott et al., 2008). Yet, the transfer to lower salinity generates a decrease in mRNA expression and protein activity of NKAα-subunit in gills of Mozambique tilapia (Lin et al., 2004). In the present study, the downregulation in *atp1a3a* expression indicates a reduction in osmoregulatory activity due the lack of salt in water.

In silverside, *slc12a2b* expression in gills and in response to increase of salinity was well detectable, evidencing the high importance of NKCC1b to hyposmoregulation of *O. humensis*. While NKA can have activity in ion absorption and secretion, NKCC1 is more associated with ion secretion in teleost gills. Here, the augmented water salt concentration leads *O. humensis* to increase its *slc12a2b* mRNA expression when FW–BW transferred. This fact, in association with the blood osmolality stabilization even when transferred to BW, indicates an efficient ion secretion activity. Furthermore, the transfer to lower salinity media did not affect the *slc12a2b* mRNA expression. Despite the opposite responses of NKCC in hypo- or hyperosmotic environment are the most common reported in fishes, none variations after salinity changes is also possible, especially in transfers for low salinities. In gills of brown trout (Tipsmark et al., 2002) and striped bass (Tipsmark et al., 2004), both gene and protein expression of NKCC1 were positively correlated to environmental salinity, with increases after FW–SW transfer and decreases after SW–FW transfer. In brackish medaka (Kang et al., 2010) and climbing perch (Loong et al., 2012), the *slc12a2b* mRNA expression and NKCC protein quantity followed the saline concentration of water inhabited by FW- and SW-acclimated fish. However, no response was reported in *slc12a2a* mRNA expression after the transfer of Mozambique tilapia from BW–FW (Breves et al., 2010).

The branchial tissue of silversides of this study had an increase in *kcnh1* mRNA expression soon after transfer to FW. A similar result was obtained in threespine stickleback, in which the *kcnh4* mRNA expression was higher in FW- than in SW-acclimated fish (Taugbøl et al., 2014). Furthermore, the return of mRNA expression to pre-transfer levels indicates an acclimation to media. Although *kcnh1* functions in organisms are not well known, our results reveal that this gene may play a role in osmoregulation.

The HSP70 overexpression is induced by some environmental stressors, such as temperature changes, UV and γ-irradiation, and chemical exposure (Place and Hofmann, 2005; Yamashita et al., 2010; Rajeshkumar et al., 2013). In *O. humensis*, the salinity change was able to induce a response of *hspa1a* gene. The same was observed in threespine stickleback (Taugbøl et al., 2014), in North Sea cod and Baltic Sea cod (Larsen et al., 2012), and in Kaluga (Peng et al., 2016). The up-regulation in *hspa1a* mRNA after BW–FW transfer indicates an increase in stress. The significant down-regulation observed after 7 days in FW indicates a reduction in stress levels, indicating acclimation. On the other hand, the unchanged *hspa1a* mRNA levels after the FW–BW transfer reveal that this salinity challenge is less stressful to *O. humensis*.

Blood glucose has been used to estimate acute stress conditions in fishes (Cataldi et al., 2005), as reported to silverside *O. bonariensis* (Tsuzuki et al., 2001). Furthermore, the hematocrit levels also can serve as stress indicator in fishes. The increment in hematocrit may indicate the presence of an active stress factor (Hudson et al., 2008). The quick increase in glycemia and the trend of increase in hematocrit, allied to the increase in *hspa1a* expression after BW–FW transfer, reveal that this “salt-free” environment is significantly more stressful to *O. humensis* than the BW medium. In *O. bonariensis*, the transfer from FW to 20 ppt BW triggered a decrease in stress followed by a decrease in glycemia and hematocrit, indicating that FW is more stressful than BW also in this species (Tsuzuki et al., 2001). Moreover, the unchanged blood glucose and hematocrit corroborates to *hspa1a* expression results, which indicates that FW–BW transfer leads to lower levels of stress in *O. humensis*.

Reduction in *atp1a3a* expression and blood osmolality in BW–FW-transferred silversides indicates a decrease in osmoregulation activity, which is energetically expensive to fishes. Even so, the stress in BW–FW group was higher, suggesting that the physiology of *O. humensis* is only partially adapted to FW. In the FW–BW transfer group, the blood osmolality maintains its levels. In *O. bonariensis*, the transfer to 20 ppt BW generates a significant increment in osmolality until 24 h post-transfer (Tsuzuki et al., 2001, 2007). The 5 ppt BW transfer also generated an increase in blood osmolality, but not as significant as 20 ppt (Tsuzuki et al., 2007). Here, *O. humensis* osmolality remained without significant variation in FW–BW. Maybe the 10 ppt salt concentration medium was insufficient to increase significantly the blood osmolality of *O. humensis*, despite the half salt concentration is able to increase this parameter of *O. bonariensis*. However, we hypothesized that FW–BW transference causes increased recruitment of both *atp1a3a* and *slc12a2b*, which would work together in the control of ion secretion and stabilization of blood osmolality. This may indicate that the silverside *O. humensis* is more efficient than *O. bonariensis* in the hyposmoregulation process.

The return of osmoregulatory genes expression and blood glucose close to pre-transfer levels after significant increase indicates an efficient acclimation (Grutter and Pankhurst, 2000; Havird et al., 2013). The blood glucose and *atp1a3a*, *slc12a2b*, *kcnh1*, and *hspa1a* mRNA stabilized in 7 days. These markers of osmoregulation and stress corroborated acclimation after 1 week despite the high stress occurred after the saline challenge.

Our results suggest that the BW–FW transfer is more stressful to *O. humensis* than the FW–BW transfer. Even though *O. humensis* is not widely cultivated in aquaculture today, the information that this species follows the responses of *O. bonariensis* and remains less stressed when in brackish environments is very important. Salinity levels were shown to modulate the energy supply available for growth and reproduction in farmed fishes (Altinok and Grizzle, 2001; Chand et al., 2015), and its optimal adjustment can benefit *O. humensis* production in captivity for aquaculture purposes. The brackish medium has potential for decrease in economic losses by mortality due to handling, transport, crowding, and poor water quality (Strüssmann et al., 1996; Tsuzuki et al., 2000b, 2001) and increase the survival rate of embryos in farm production (Piedras et al., 2009). Keeping *O. humensis* in this near-isosmotic environment can potentially allow for better growth rates, food conversion ratio, energy absorption efficiency, among other parameters of interest. Furthermore, the cultivation of *O. humensis* in conditions closer to the ideal decreases the stress and the physiological variations due to it and favors the use of this species as a model in scientific research.

Even regarded as FW, these species are commonly in contact with salt and BW on estuary and coastal lagoons of South America and have better development and survival in saline environments (Tsuzuki et al., 2000a, 2001; Piedras et al., 2009). The coastal plain of southern Brazil system was originated from successive transgressions and regressions moves since the upper Pleistocene (Bemvenuti, 2006). The formation and radiation of *Odontesthes* spp. is also recent and occurs during Pleistocene–Holocene (Beheregaray et al., 2002; Lovejoy et al., 2006; Heras and Roldán, 2011). Furthermore, there are strong evidences that various genus of Atherinopsidae order, including *Odontesthes*, have an evolutionary tendency to invade the continental FWs (Bloom et al., 2013; Campanella et al., 2015). Thus, the same events that originated the Southeastern South America continental lakes and lagoons may have triggered the speciation process of *Odontesthes* spp.

The previous and the present study corroborate to the theory that the FW is not the ideal environment for *O. humensis* and give more arguments to the theory of repeated invasion of FW habitats by silversides marine ancestors. The conquest of the FW environment is very recent, so there has not been enough time for the selection of adaptive mechanisms for life in this condition without the elimination of some basal stress levels.

## Author Contributions

TS, GM, and VC were responsible for experimental design, data analysis, and manuscript writing. TS, GM, MR, and RR were responsible for fish acclimation and maintenance. TS, GM, BB, IL, LS, MR, RR, and WD were responsible for the biological collections. TS, BB, IL, LS, WD, and VC were responsible for the molecular biology, from RNA extraction to sequencing, and qRT-PCR analysis. OD, TC, and FS were also responsible for qRT-PCR analysis. DP and RR were also responsible for data analysis and language review.

## Conflict of Interest Statement

The authors declare that the research was conducted in the absence of any commercial or financial relationships that could be construed as a potential conflict of interest.

## Footnotes

1 http://blast.ncbi.nlm.nih.gov/

2 https://www.expasy.org/

3 http://www.uniprot.org/

4 http://primer3.ut.ee

## Supplementary Material

FIGURE S1 Phylogenetic analysis of *NKAα*-subunit amino acid sequences of mammals and teleosts. The tree was generated by MEGA v6 software using the Neighbor-Joining method. The bootstrap values from 10,000 replicates are showed at each node. Values between parentheses represent the identity (in %) of the sequence with *NKAα3a* of *Odontesthes humensis*. Scale bar units represent the number of amino acid substitutions per site. *NKAα1* GenBank ID: *Homo sapiens*, CAA27840.1. *NKAα1a* GenBank ID: *Danio rerio*, NP_571762.1; *Solea senegalensis*, BAN17690.1; *Fundulus heteroclitus*, AAL18002.1; *Oncorhynchus masou*, BAJ13363.1; *Oncorhynchus mykiss*, NP_001117933.1. *NKAα1b* GenBank ID: *O. mykiss*, NP_001117932.1; *O. masou*, BAJ13362.1; *S. senegalensis*, BAN17691.1; *D. rerio*, NP_571765.1. *NKAα2* GenBank ID: *Rattus norvegicus*, NP_036637.1; *O. mykiss*, NP_001117930.1; *D. rerio*, AAF98359.1; *S. senegalensis*, BAO02373.1; *F. heteroclitus*, AAL18003.1. *NKAα3* GenBank ID: *H. sapiens*, NP_689509.1; *R. norvegicus*, NP_036638.1; *Oreochromis mossambicus*, AAF75108.1; *O. mykiss*, NP_001118102.1. *NKAα3a* GenBank ID: *D. rerio*, NP_571759.2; *S. senegalensis*, BAN17693.1; *Monopterus albus*, AGV06213.1. *NKAα3b* GenBank ID: *M. albus*, AGV06214.1; *S. senegalensis*, BAN17692.1. *NKAα4* GenBank ID: *H. sapiens*, Q13733.3; *R. norvegicus*, NP_074039.1. *NKAα1* from *Caenorhabditis elegans*, NP_506269.1, was used as outgroup.

FIGURE S2 Phylogenetic analysis of *NKCC* amino acid sequences of mammals and teleosts. The tree was generated by MEGA v6 software using the Neighbor-Joining method. The bootstrap values from 10,000 replicates are showed at each node. Values between parentheses represent the identity (in %) of the sequence with *NKCC1b* of *O. humensis*. Scale bar units represent the number of amino acid substitutions per site. *NKCC1* GenBank ID: *H. sapiens*, P55011.1; *Mus musculus*, NP_033220.2. *NKCC1a* GenBank ID: *Anguilla Anguilla*, CAD31111.1; *Salmo salar*, NP_001117155.1; *Oryzias dancena*, ADN18710.1; *O. mossambicus*, AAR97731.1; *Anabas testudineus* AFK29496.1. *NKCC1b* GenBank ID: *O. dancena*, ADK47392.1; *M. albus*, AGX01628.1; *O. mossambicus*, AAR97732.1; *A. anguilla*, CAD31112.1. *NKCC2* GenBank ID: *H. sapiens*, Q13621.2; *M. musculus*, P55014.2; *O. mossambicus*, AAR97733.1; *Takifugu obscurus*, BAH20440.1. *NKCC1* from *Strongylocentrotus purpuratus*, NP_001106707.1, was used as outgroup.

FIGURE S3 Phylogenetic analysis of *KCNH* amino acid sequences from EAG and ELK subfamilies of mammals and teleosts. Third existent ERG subfamily was discard in order to maintain an abbreviated analysis. The tree was generated by MEGA v6 software using the Neighbor-Joining method. The bootstrap values from 10,000 replicates are showed at each node. Values between parentheses represent the identity (in %) of the sequence with *KCNH1* of *O. humensis*. Scale bar units represent the number of amino acid substitutions per site. *KCNH1* GenBank ID: *H. sapiens*, NP_758872.1; *M. musculus*, NP_034730.1; *D. rerio*, XP_009291371.1; *Oreochromis niloticus*, XP_005474450.1. *KCNH3* GenBank ID: *H. sapiens*, NP_001300959.1; *D. rerio*, XP_001919436.3; *Nothobranchius furzeri*, SBP57798.1. *KCNH4* GenBank ID: *H. sapiens*, NP_036417.1; *P. reticulata*, XP_008415281.2; *O. niloticus*, XP_019213276.1; *Maylandia zebra*, XP_012774834.1. *KCNH5* GenBank ID: *H. sapiens*, NP_647479.2; *M. musculus*, NP_766393.2; *D. rerio*, NP_001263209.1; *O. niloticus*, XP_003451242.1. *KCNH8* GenBank ID: *H. sapiens*, NP_653234.2; *M. musculus*, NP_001026981.2; *O. niloticus*, XP_003448945.1; *M. zebra*, XP_004547937.1; *Poecilia reticulata*, XP_008419899.1. *KCNH1* from *Toxocara canis*, KHN74999.1, was used as outgroup.

FIGURE S4 Phylogenetic analysis of *HSP70* amino acid sequences of mammals and teleosts. The tree was generated by MEGA v6 software using the Neighbor-Joining method. The bootstrap values from 10,000 replicates are showed at each node. Values between parentheses represent the identity (in %) of the sequence with *HSP70* of *Odontesthes humensis*. Scale bar units represent the number of amino acid substitutions per site. *HSP70-1A* GenBank ID: *H. sapiens*, NP_005336.3; *M. musculus*, NP_034609.2; *Notothenia coriiceps*, XP_010769991.1; *M. albus*, AGO01980.1; *Nothobranchius korthausae*, SBQ68887.1. *HSP70-1B* GenBank ID: *M. albus*, AGO01981.1; *D. rerio*, NP_001093532.1. *HSP70-2* GenBank ID: *H. sapiens*, AAH36107.1; *M. musculus*, EDL36460.1. *HSP70-1* from *C. elegans*, NP_503068.1, was used as outgroup.

TABLE S1 Expression of reference genes of *Odontesthes humensis* before (D0) and after (D1, D7, and D15) hypo– and hyperosmotic shock. Equal letters represent no difference in a same column. Abbreviations: Ct, cycle threshold values; D0, day zero; D1, day one; D7, day seven; D15, day fifteen; FW–BW, transfer from freshwater to brackish water; BW–FW, transfer from brackish water to freshwater.

## Abbreviations

BW: brackish water
FW: freshwater
SW: seawater
