# Corrigendum: Gene and Blood Analysis Reveal That Transfer from Brackish Water to Freshwater Is More Stressful to the Silverside *Odontesthes humensis*

**DOI:** 10.3389/fgene.2019.00250

**Published:** 2019-03-26

**Authors:** Tony L. R. Silveira, Gabriel B. Martins, William B. Domingues, Mariana H. Remião, Bruna F. Barreto, Ingrid M. Lessa, Lucas Santos, Danillo Pinhal, Odir A. Dellagostin, Fabiana K. Seixas, Tiago Collares, Ricardo B. Robaldo, Vinicius F. Campos

**Affiliations:** ^1^Laboratory of Structural Genomics, Technological Development Center, Federal University of Pelotas, Pelotas, Brazil; ^2^Laboratory of Physiology, Institute of Biology, Federal University of Pelotas, Pelotas, Brazil; ^3^Laboratory of Cancer Biotechnology, Technological Development Center, Federal University of Pelotas, Pelotas, Brazil; ^4^Genomics and Molecular Evolution Laboratory, Department of Genetics, Institute of Biosciences of Botucatu, São Paulo State University, Botucatu, Brazil; ^5^Laboratory of Vaccinology, Technological Development Center, Federal University of Pelotas, Pelotas, Brazil

**Keywords:** acclimation, blood, brackish water, fish, freshwater, genes, salt, transfer

In the original article, there was an error. The affirmation present in the title does not correspond to the main conclusion of the study. In the body text, all ideas expressed are correct. During the editing process, a change in the title was requested. It was then changed with an error independent of the main text. Thus, the mistaken title was published as follows: “Gene and Blood Analysis Reveal That Transfer from Brackish Water to Freshwater Is Less Stressful to the Silverside *Odontesthes humensis*”. In contrast to that, our results suggest that brackish water (BW) to freshwater (FW) transfer is more stressful to *O. humensis* than FW-BW transfer. The alteration of a single word of the title solves the problem.

A correction has therefore been made to the title:

“Gene and Blood Analysis Reveal That Transfer from Brackish Water to Freshwater Is More Stressful to the Silverside *Odontesthes humensis*.”

The authors apologize for this error and state that this does not change the scientific conclusions of the article in any way. The original article has been updated.

