# Supplementary figures and images for: Gene and Blood Analysis Reveal That Transfer from Brackish Water to Freshwater Is More Stressful to the Silverside Odontesthes humensis
Source: Front Genet. 2018 Feb 6;9:28. doi: 10.3389/fgene.2018.00028 (PMC5836595; doi:10.3389/fgene.2018.00028)

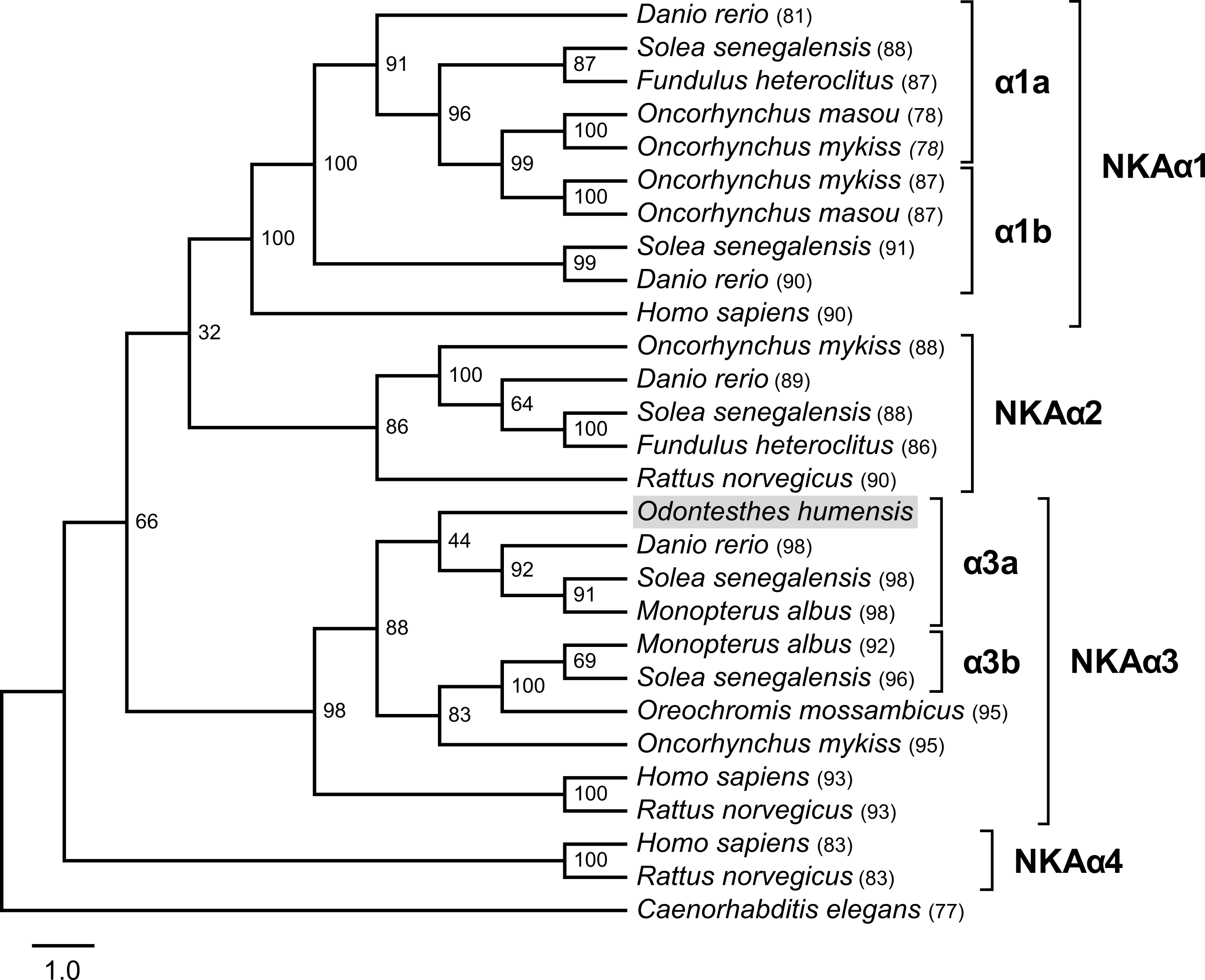

Supplement: FIGURE S1 — Phylogenetic analysis of NKAα-subunit amino acid sequences of mammals and teleosts. The tree was generated by MEGA v6 software using the Neighbor-Joining method. The bootstrap values from 10,000 replicates are showed at each node. Values between parentheses represent the identity (in %) of the sequence with NKAα3a of Odontesthes humensis. Scale bar units represent the number of amino acid substitutions per site. NKAα1 GenBank ID: Homo sapiens, CAA27840.1. NKAα1a GenBank ID: Danio rerio, NP_571762.1; Solea senegalensis, BAN17690.1; Fundulus heteroclitus, AAL18002.1; Oncorhynchus masou, BAJ13363.1; Oncorhynchus mykiss, NP_001117933.1. NKAα1b GenBank ID: O. mykiss, NP_001117932.1; O. masou, BAJ13362.1; S. senegalensis, BAN17691.1; D. rerio, NP_571765.1. NKAα2 GenBank ID: Rattus norvegicus, NP_036637.1; O. mykiss, NP_001117930.1; D. rerio, AAF98359.1; S. senegalensis, BAO02373.1; F. heteroclitus, AAL18003.1. NKAα3 GenBank ID: H. sapiens, NP_689509.1; R. norvegicus, NP_036638.1; Oreochromis mossambicus, AAF75108.1; O. mykiss, NP_001118102.1. NKAα3a GenBank ID: D. rerio, NP_571759.2; S. senegalensis, BAN17693.1; Monopterus albus, AGV06213.1. NKAα3b GenBank ID: M. albus, AGV06214.1; S. senegalensis, BAN17692.1. NKAα4 GenBank ID: H. sapiens, Q13733.3; R. norvegicus, NP_074039.1. NKAα1 from Caenorhabditis elegans, NP_506269.1, was used as outgroup. [file Image_1.TIFF]

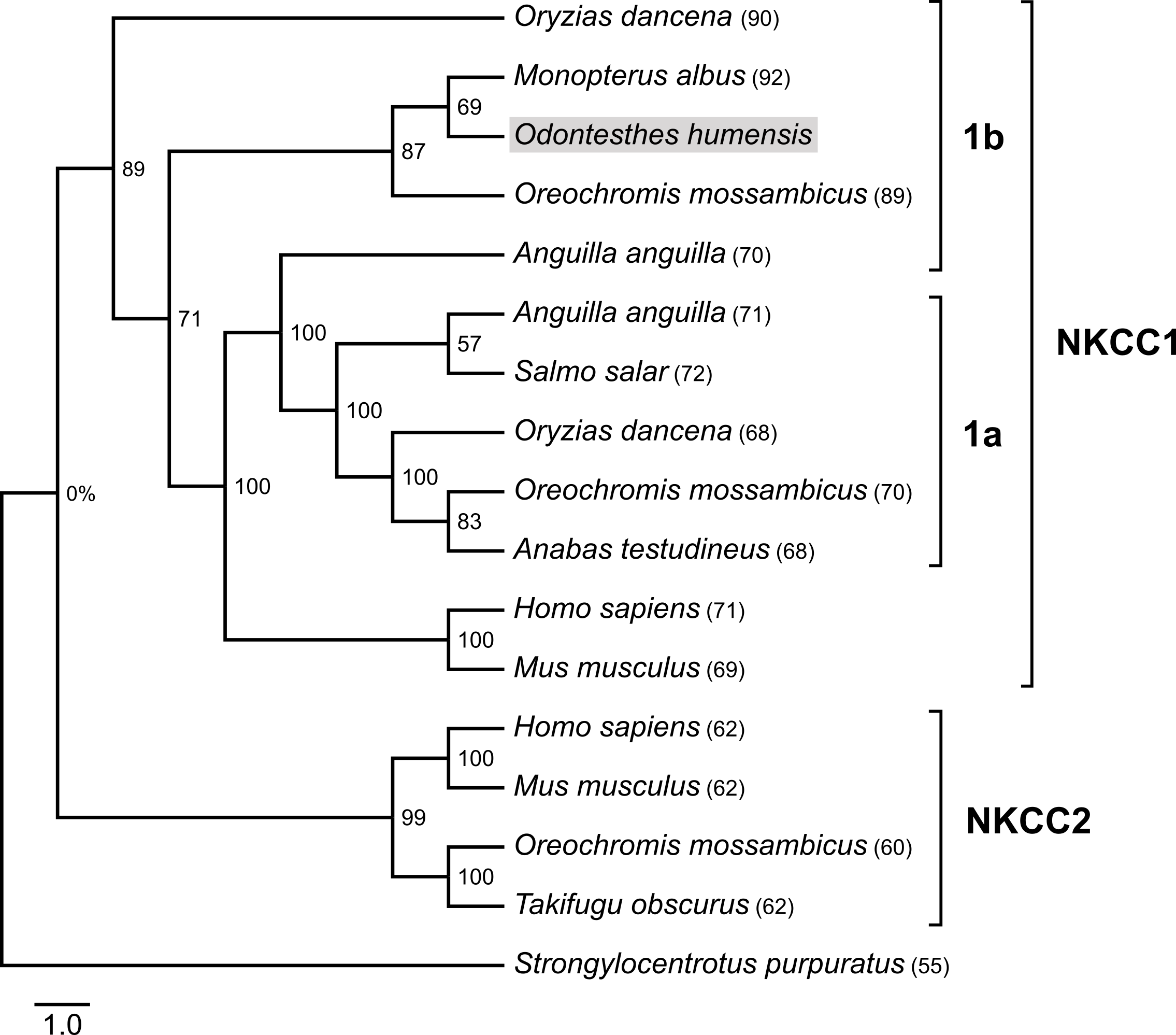

Supplement: FIGURE S2 — Phylogenetic analysis of NKCC amino acid sequences of mammals and teleosts. The tree was generated by MEGA v6 software using the Neighbor-Joining method. The bootstrap values from 10,000 replicates are showed at each node. Values between parentheses represent the identity (in %) of the sequence with NKCC1b of O. humensis. Scale bar units represent the number of amino acid substitutions per site. NKCC1 GenBank ID: H. sapiens, P55011.1; Mus musculus, NP_033220.2. NKCC1a GenBank ID: Anguilla Anguilla, CAD31111.1; Salmo salar, NP_001117155.1; Oryzias dancena, ADN18710.1; O. mossambicus, AAR97731.1; Anabas testudineus AFK29496.1. NKCC1b GenBank ID: O. dancena, ADK47392.1; M. albus, AGX01628.1; O. mossambicus, AAR97732.1; A. anguilla, CAD31112.1. NKCC2 GenBank ID: H. sapiens, Q13621.2; M. musculus, P55014.2; O. mossambicus, AAR97733.1; Takifugu obscurus, BAH20440.1. NKCC1 from Strongylocentrotus purpuratus, NP_001106707.1, was used as outgroup. [file Image_2.TIFF]

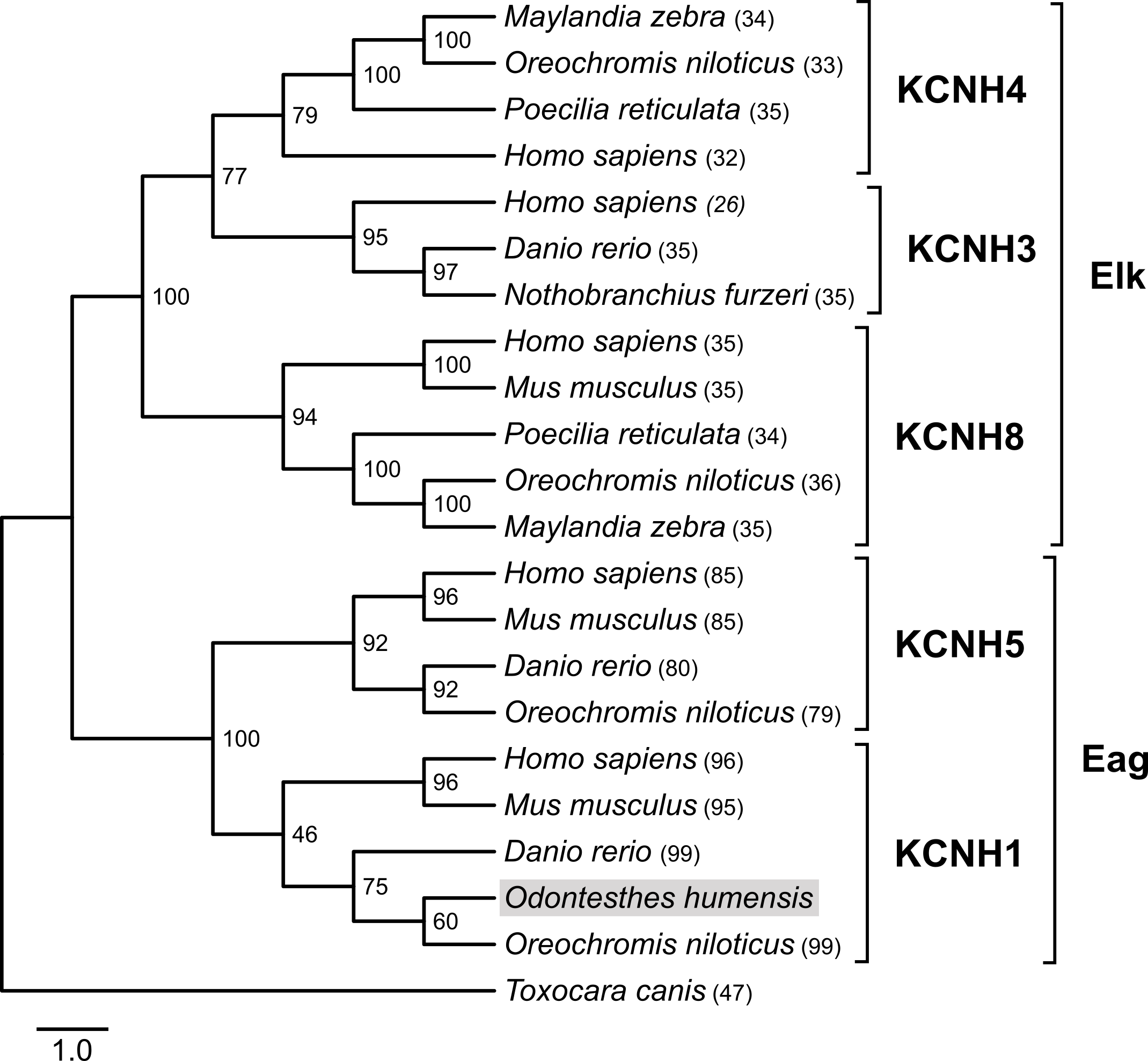

Supplement: FIGURE S3 — Phylogenetic analysis of KCNH amino acid sequences from EAG and ELK subfamilies of mammals and teleosts. Third existent ERG subfamily was discard in order to maintain an abbreviated analysis. The tree was generated by MEGA v6 software using the Neighbor-Joining method. The bootstrap values from 10,000 replicates are showed at each node. Values between parentheses represent the identity (in %) of the sequence with KCNH1 of O. humensis. Scale bar units represent the number of amino acid substitutions per site. KCNH1 GenBank ID: H. sapiens, NP_758872.1; M. musculus, NP_034730.1; D. rerio, XP_009291371.1; Oreochromis niloticus, XP_005474450.1. KCNH3 GenBank ID: H. sapiens, NP_001300959.1; D. rerio, XP_001919436.3; Nothobranchius furzeri, SBP57798.1. KCNH4 GenBank ID: H. sapiens, NP_036417.1; P. reticulata, XP_008415281.2; O. niloticus, XP_019213276.1; Maylandia zebra, XP_012774834.1. KCNH5 GenBank ID: H. sapiens, NP_647479.2; M. musculus, NP_766393.2; D. rerio, NP_001263209.1; O. niloticus, XP_003451242.1. KCNH8 GenBank ID: H. sapiens, NP_653234.2; M. musculus, NP_001026981.2; O. niloticus, XP_003448945.1; M. zebra, XP_004547937.1; Poecilia reticulata, XP_008419899.1. KCNH1 from Toxocara canis, KHN74999.1, was used as outgroup. [file Image_3.TIFF]

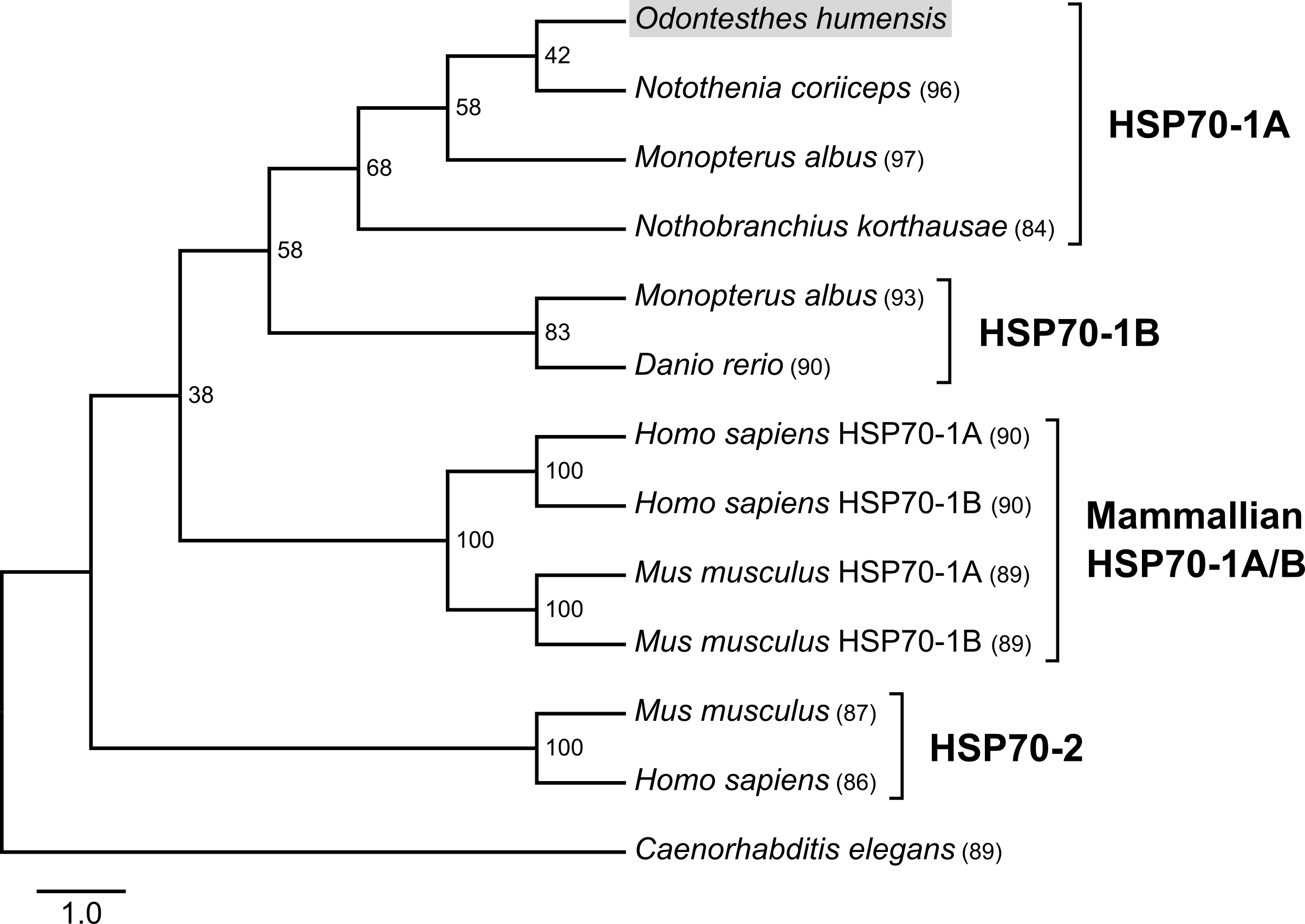

Supplement: FIGURE S4 — Phylogenetic analysis of HSP70 amino acid sequences of mammals and teleosts. The tree was generated by MEGA v6 software using the Neighbor-Joining method. The bootstrap values from 10,000 replicates are showed at each node. Values between parentheses represent the identity (in %) of the sequence with HSP70 of Odontesthes humensis. Scale bar units represent the number of amino acid substitutions per site. HSP70-1A GenBank ID: H. sapiens, NP_005336.3; M. musculus, NP_034609.2; Notothenia coriiceps, XP_010769991.1; M. albus, AGO01980.1; Nothobranchius korthausae, SBQ68887.1. HSP70-1B GenBank ID: M. albus, AGO01981.1; D. rerio, NP_001093532.1. HSP70-2 GenBank ID: H. sapiens, AAH36107.1; M. musculus, EDL36460.1. HSP70-1 from C. elegans, NP_503068.1, was used as outgroup. [file Image_4.TIFF]
