# Supplementary material for: Gene and Blood Analysis Reveal That Transfer from Brackish Water to Freshwater Is More Stressful to the Silverside Odontesthes humensis
Source: Front Genet. 2018 Feb 6;9:28. doi: 10.3389/fgene.2018.00028 (PMC5836595; doi:10.3389/fgene.2018.00028)
Supplement: TABLE S1 — Expression of reference genes of Odontesthes humensis before (D0) and after (D1, D7, and D15) hypo– and hyperosmotic shock. Equal letters represent no difference in a same column. Abbreviations: Ct, cycle threshold values; D0, day zero; D1, day one; D7, day seven; D15, day fifteen; FW–BW, transfer from freshwater to brackish water; BW–FW, transfer from brackish water to freshwater. [file Table_1.pdf]

Supplementary Table 1: Expression of reference genes of *Odontesthes humensis* before (D0) and after (D1, D7 and D15) hypo and hyperosmotic shock. Equal letters represent no difference in a same column.

**Treatment**	**Time points**	**Ct values of reference genes**	
		actb	*h3a*
FW-BW transfer	D0	24.90 (±0.69) [a]	20.43 (±0.77) [a]
	D1	24.56 (±0.55) [a]	20.32 (±0.49) [a]
	D7	24.53 (±0.77) [a]	20.02 (±0.68) [a]
	D15	24.55 (±0.74) [a]	20.00 (±0.71) [a]
BW-FW transfer	D0	24.53 (±0.90) [a]	20.51 (±1.05) [a]
	D1	24.25 (±0.59) [a]	20.36 (±0.52) [a]
	D7	24.98 (±0.40) [a]	20.24 (±0.50) [a]
	D15	25.00 (±0.99) [a]	21.00 (±1.29) [a]

Abbreviations: Ct, cycle threshold values; D0, day zero; D1, day one; D7, day seven; D15, day fifteen; FW-BW, transfer from freshwater to brackish water; BW-FW, transfer from brackish water to freshwater.
